# Effect of temozolomide on livin and caspase-3 in U251 glioma stem cells

**DOI:** 10.3892/etm.2014.2144

**Published:** 2014-12-18

**Authors:** GENHUA LI, HAO ZHANG, YANG LIU, LINGSHENG KONG, QIANG GUO, FENG JIN

**Affiliations:** Neuro-oncology Laboratory, Department of Neurosurgery, Affiliated Hospital of Jining Medical College, Jining, Shandong 272029, P.R. China

**Keywords:** glioma, stem cells, livin, caspase-3, cell cycle, temozolomide

## Abstract

The aim of the present study was to analyze the effect of temozolomide (TMZ) on the antiapoptotic gene livin and the associated gene caspase-3. Cancer stem cells were isolated from U251 glioblastoma cells using immunomagnetic beads. The glioma cells and glioma stem cells were transfected with livin or small hairpin RNA (shRNA) against livin using lentiviral vectors. Quantitative PCR, flow cytometry and a Cell Counting kit-8 assay were used to detect the expression of livin and caspase-3, analyze the cell cycle and investigate cell proliferation, respectively, following treatment with various concentrations of TMZ (0, 25, 50, 100, 200 and 400 μmol/l) for different periods of time (24, 48 and 72 h). The expression levels of livin and caspase-3 in the U251 stem cells were significantly higher than those in the U251 cells (P<0.01). At the same intervention time, the expression levels of livin decreased and those of caspase-3 increased as the concentration of TMZ increased (P<0.05). The expression levels of livin and caspase-3 in the U251 cells were lower than those in the U251 stem cells with the same intervention time and concentration of TMZ (P<0.05). The cell cycle was arrested in the G2/M phase in the U251 cells following TMZ intervention; the proportion of cells in the G2/M phase increased as the concentration of TMZ increased (P<0.05). The U251 stem cells were arrested in the S phase following treatment with TMZ; the proportion of cells in the S phase increased as the concentration of TMZ increased (P<0.05). In conclusion, the expression levels of livin and caspase-3 were effectively inhibited and increased, respectively, in all cell models following treatment with TMZ. TMZ is able to arrest the cell cycle and enhance cell apoptosis. U251 stem cells are less vulnerable than U251 cells to TMZ.

## Introduction

Glioblastoma is the most common and most devastating type of primary brain tumor. At present, the treatment of glioblastoma involves surgery, radiotherapy and chemotherapy, all of which are acknowledged as palliative measures, meaning that they do not provide a cure, and the median survival time for patients with glioblastoma multiforme is only ~14.6 months (1,2). On this basis, it is necessary to find new methods to improve the treatment of glioblastoma.

There is compelling evidence that cancer stem cells play a key role in cancer drug resistance, occurrence and development; CD133^+^ cells are regarded as having self-renewal and infinite proliferation abilities (3). Based on the concept of cancer stem cells, a new mode of tumor resistance has been identified. The natural resistance mechanism of cancer stem cells, including the adoption of a resting state, the ability to repair DNA, the expression of ABC transporters and resistance to apoptosis, all lead to stem cells remaining following chemotherapy (4). It has been found that only 100 CD133^+^ stem cells are required to successfully establish a new glioma when serially transplanted (5). The biological characteristics of cancer stem cells may led to the failure of long-term chemotherapy; the overexpression of multidrug resistance proteins (MRPs) has been observed in cancer stem cells isolated from certain solid tumors. MRPs provide tumor progenitor cells with resistance to the killing effect of cytotoxic drugs and alter the differentiation of cells (6). Therefore, the study of glioma stem cells may be a key step to solving the problem of tumor chemotherapy failure and tumor recurrence.

The present study investigated the expression of livin in glioma cells, including glioma stem cells. Temozolomide (TMZ) intervention in a cell model with lentivirus transfection was used to investigate the changes in the expression of livin and the associated caspase-3 in U251 glioma cells and U251 stem cells. The effects on the cell cycle of the changes in livin expression and TMZ intervention were also examined.

## Materials and methods

### Chemicals and reagents

Dulbecco’s modified Eagle’s medium/Nutrient Mixture F-12 Ham’s (DMEM/F12) and fetal bovine serum (FBS) were purchased from HyClone (Logan, UT, USA). B-27 (50X) Serum-Free Supplement was from Gibco (Grand Island, NY, USA). Epidermal growth factor (EGF) and basic fibroblast growth factor (bFGF) were obtained from Peprotech (Rocky Hill, NJ, USA). Leukemia inhibitory factor (LIF) was obtained from ProSpec-Tany TechnoGene Ltd. (Rehovot, Israel). The CD133 cell isolation kit [magnetic cell sorting (MACS) method] was purchased from Miltenyi Biotec GmbH (Bergisch Gladbach, Germany). Antibodies to nestin, glial fibrillary acidic protein (GFAP) and tubulin-β were obtained from Santa Cruz Biotechnology, Inc. (Santa Cruz, CA, USA). Cell Counting kit-8 (CCK-8) was obtained from Dojindo Molecular Technologies, Inc. (Kumamoto, Japan). Cell Cycle and Apoptosis Analysis kits and trypsin were obtained from Beyotime Institute of Biotechnology (Shanghai, China). Lentivirus was purchased from Shanghai GeneChem Co., Ltd. (Shanghai, China).

### Glioma cell line culture

The U251 glioblastoma cell line was provided by China Center for Typical Culture Collection (CCTCC, Wuhan, China). The cell line was cultured in a medium containing DMEM/F12, 10% FBS, 100 U/ml benzylpenicillin and 100 μg/ml streptomycin, under conditions of 37°C, 5% CO_2_ and saturated humidity.

### Isolation and identification of CD133^+^ glioma stem cells

The U251 cells were collected and inoculated at low density into a serum-free medium [neural stem cell (NSC) medium] that contained DMEM/F12, 20 ng/ml EGF, 20 ng/ml bFGF, 10 ng/ml LIF and B-27 (1X). The cells were placed in an incubator under conditions of 37°C, 5% CO_2_ and saturated humidity. Once every 3–4 days, half of the medium was replaced. After the neurospheres had grown in large quantities, the spheres were collected and CD133^+^ cells were separated by a MACS technique. The sorting process was conducted according to the instructions of the CD133 cell isolation kit.

The well-grown cell spheres were selected for growing on polylysine-coated slides. After drying at 37°C, the slides were washed with phosphate-buffered saline (PBS) three times in order to clear away the medium. At room temperature, the cells were fixed with paraformaldehyde for 30 min, and then washed with PBS again three times. After blocking with 5% goat serum at 37°C for 30 min, rabbit anti-human nestin (1:200; primary antibody) was added and the cells were placed in a wet box overnight. The day following PBS washing, goat anti-rabbit IgG-FITC antibody (secondary antibody) was added for incubation for 30 min at 37°C. In addition, a negative control assay in which PBS was used instead of the primary antibody was performed. The slides were observed with an Olympus IX71 fluorescence microscope (Olympus, Tokyo, Japan). The immunofluorescence assay procedures for the detection of GFAP and β-tubulin on differentiated glioma stem cells were as described for nestin, with the exception that respective primary antibodies were used.

### Cell morphology observation

After the TMZ (0, 25, 50, 100, 200 or 400 μmol/l) had been added to the cells for 48 h, the cell morphology was observed with an inverted microscope (Olympus CKX41; magnification, ×10).

### Transfection with lentivirus

Cells (1×10^5^) were inoculated into 6-well plates. According to the instructions of the lentivirus transfection reagent, and with multiplicities of infection (MOI) determined in a preliminary experiment (U251 cells, MOI=5; U251 stem cells, MOI=10), lentivirus encoding livin or small hairpin RNA (shRNA) against livin was directly mixed with the enhanced infection solution (slow virus diluent), and then mixed with 500 μl culture medium under conditions of 37°C, 5% CO_2_ and saturated humidity. After 20 h of contact with the cells, the lentivirus medium was replaced with ordinary medium. Three days later, lentiviral transfection was observed under a fluorescence microscope. The transfection procedures were conducted using biological safety equipment.

### CCK-8 assay for cell survival analysis

Following treatment with the various concentrations of TMZ for 48 h, the cell survival rate was determined using the CCK-8 solution according to the manufacturer’s instructions. Cells in 96-well plate were treated with 10 μl CCK-8 solution, and incubated for 2 h at 37°C. The absorbance (A) of each well was quantified at 450 nm using an automated ELISA reader (Bio-Tek Instruments Inc., Winooski, VT, USA). The cell survival rate (%) was calculated as follows: [A(experimental well) − A(blank well)]/[A(control well) − A(blank well)] × 100.

### Cell cycle assay by flow cytometry

Following treatment with the various concentrations of TMZ for 48h, the cell cycles of the U251 cells and U251 stem cells were determined by flow cytometry. Briefly, the culture medium was collected, and the treated cells were digested with 0.05% trypsin for 3–5 min. The digested cells were washed with phosphate-buffered saline, and then fixed in 70% ethyl alcohol overnight. Then, ~1×10^6^ cells were incubated with the cell cycle detection kit according to the manufacturer’s instructions prior to analysis by flow cytometry (BD^™^ LSR II; BD Biosciences, Franklin Lanes, NJ, USA). Control cells (transfected with an empty vector) were similarly processed.

### Quantitative PCR (qPCR)

Following treatment of the U251 and U251 stem cells with the various concentrations of TMZ for 48 h, or with 400 μmol/l TMZ for various times (0, 24, 48 and 72h), qPCR was performed as described in a previous study by the authors (7). The cell samples (1×10^6^) were collected and combined with 1 ml TRIzol reagent (Invitrogen Life Technologies, Carlsbad, CA, USA) according to manufacturer’s instructions, in order to obtain the total RNA from the U251 cells and U251 stem cells. The RNA solution was stored at −80°C until used. All reactions were performed in duplicate with a negative control (no template) and the mean threshold cycle value (the start of exponential amplification) of each sample was normalized with the threshold cycle value of glyceraldehyde-3-phosphate dehydrogenase (GAPDH), to provide the ΔCt value. qPCR was performed using a 7900HT Sequence Detection system (Applied Biosystems, Foster City, CA, USA). Reverse transcription was performed with M-MLV Reverse Transcriptase (Takara Bio, Inc., Shiga, Japan). The reverse transcriptional reaction system including 5.5 μl H_2_O, 1.0 μl oligo(dT)_18_ (50 μg/ml) and 6.0 μl total RNA was heated to 70°C for 5 min and then chilled with ice to unfold the mRNA secondary structure; and the subsequent step included 0.5 μl RNasin (40 U/μl), 4.0 μl 5X buffer, 2.0 μl dNTP (10 mM) and 1.0 μl RTase (200 U/μl), with heating to 42°C for 60 min, 95°C for 5 min and chilling to 4°C. The qPCR reaction was performed with SYBR-Green I fluorochrome. A standard curve was obtained and the cycle threshold (Ct) value was calculated.

Each 50 μl PCR system contained 1/50 of the original cDNA, 7 μl (25 mM) MgCl_2_, 0.8 μl (20 pmol/μl) each primer, 1 μl (10 mM) dNTP, 1 μl SYBR-Green I, 0.5 μl (5 U/μl) Taq DNA polymerase (Promega Corporation, Madison, WI, USA) and 5 μl 10X buffer. Fifty cycles of amplification were performed: 94°C for 30 sec, 57°C for 30 sec, then 72°C for 30 sec. The fluorescence signal was detected at the end of each cycle. Melting curve analysis was used to confirm the specificity of the products. The 2^−ΔΔCT^ method was used to analyze the results (8). The primers were as follows: homo-livin, forward: 5′-GCTGTCAGTTCCTGCTCCGGTC-3′ and reverse: 5′-CAGGGGCTGCGTCTTCCGGTTC-3′; homo-caspase-3, forward: 5′-GAAGCGAATCAATGGACTCTGG-3′ and reverse: 5′-GTTTGCTGCATCGACATCTGTAC-3′.

### Statistical analysis

Each test was performed in triplicate. The results are presented as mean ± standard deviation. Comparisons of the data were performed with Student’s t-test and one-way analysis of variance. P<0.05 was considered to indicate a statistically significant difference. The statistical analysis was performed with SPSS software, version 13.0 (SPSS, Inc., Chicago, IL, USA).

## Results

### Cell morphology

**Figure 1:**
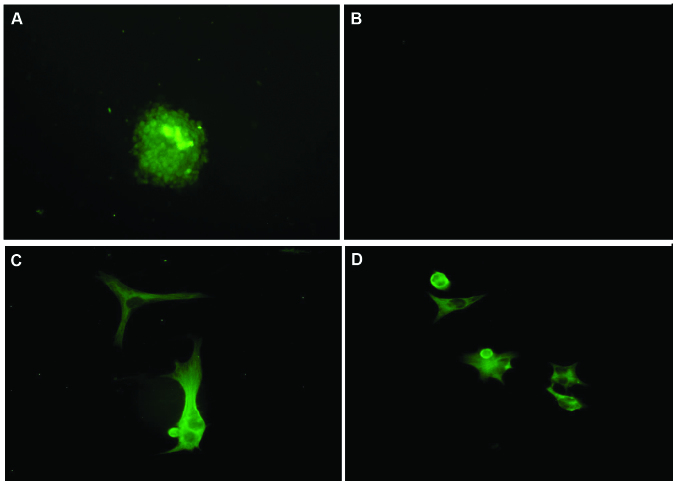
(A) Stem cell spheres were stained nestin positive. (B) Negative control stem cell spheres. Stem cell spheres were induced after 7 days in serum medium and were stained (C) GFAP positive and (D) β-tubulin positive (all magnification, ×20).

CD133^+^ cells were successfully separated from U251 glioma cells by an immunomagnetic bead technique. The stem cells began to grow together and form cell spheres after 3 days in the NCS medium. The cells tested positive for nestin. The stem cell spheres were induced to break up after 7 days in serum medium, and diverse cell morphology was observed: the cells were attached, triangular, rounded or irregular in form, with elongated cell bodies, and stained positive for GFAP and β-tubulin. These observations confirmed that the stem cells had been induced to differentiate into neural cells (Fig. 1).

Under an inverted microscope, following 48 h of TMZ intervention, U251 cell and U251 stem cell proliferation was inhibited. For the U251 cells, cell death was observed; the cell density was significantly lower than that of the control group and cell shrinkage, cell nucleus disintegration and the presence of cell fragments floating on the medium surface were observed. For the U251 stem cells, the morphology of the stem cell spheres changed, the cells became gray and breakdown of the nucleus was observed, but no cell fragments were present.

### Cell survival

**Figure 2:**
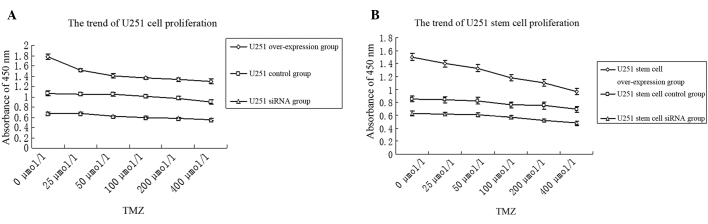
Proliferation trends of post-transfected cells that were cultured for 7 days. The cells were implanted in 6-well and 96-well plates with different concentrations of TMZ (0, 25, 50, 100, 200 and 400 μmol/l; at 0 μmol/l, TMZ was replaced by DMSO) and cultured for 48 h. (A) U251 cells and (B) U251 stem cells. TMZ, temolozomide.

A CCK-8 kit was used to detect the cell activity of the U251 cells and stem cells following TMZ intervention for 48 h. The degree of proliferation of the U251 cells and U251 stem cells at the same concentration and intervention time was as follows: livin-overexpressing group > control group > livin-shRNA group. It was observed that the adherent U251 cells proliferated faster than the U251 tumor stem cells in the presence of the same TMZ concentration for the same treatment time. TMZ inhibited the cell proliferation of livin-overexpressing U251 cells and U251 stem cells (P<0.05; Fig. 2).

### qPCR results

qPCR demonstrated that there were higher expression levels of livin and caspase-3 in the U251 glioma stem cells than in the U251 glioma cells. TMZ effectively inhibited the expression of livin in the U251 cells and U251 stem cells in all cell models (P<0.05). The expression levels of livin were reduced as the concentration of TMZ increased (Table I). The levels of caspase-3 tended to increase as the concentration of TMZ increased (Table II). When the same concentration of TMZ was used, the expression levels of livin and caspase-3 were reduced and increased, respectively, as the treatment time was prolonged (Table III). The overexpression of livin and low expression of caspase-3 may enhance the proliferation of cells, including that of stem cells. TMZ increased the expression of caspase-3 and downregulated the expression of livin both in U251 cells and stem cells.

### Cell cycle

In the U251 cell group, compared with the respective transfected control group, the livin-overexpressing group had an increased proportion of cells in the G2-M phase, whereas the livin-shRNA group had marked increases in the proportions of cells in the S and G2-M phases (P<0.05). Following 48 h of exposure to 400 μmol/l TMZ, the control and livin-shRNA groups demonstrated increases in the proportions of cells in the G2-M phase, whereas the livin-overexpressing group had an increased proportion of cells in the S phase (P<0.05). These results indicate that the effect of TMZ on the cell cycle was different from that on the blank control group following transfection with the two lentiviruses.

**Figure 3:**
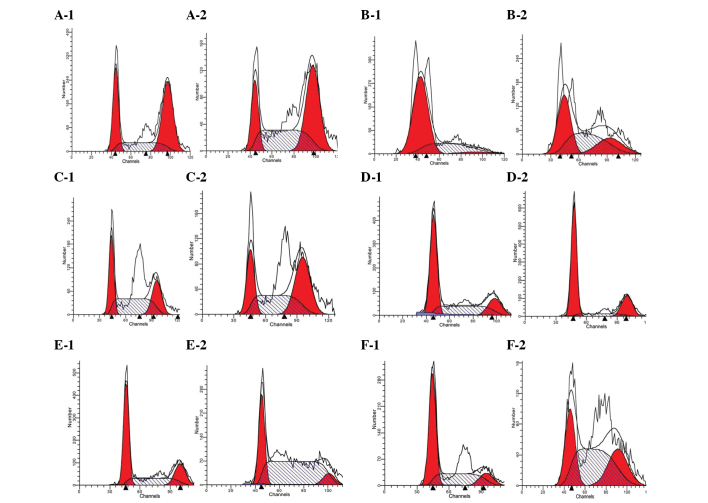
(A-1) U251 livin-overexpressing cells; (A-2) U251 livin-overexpressing cells treated with TMZ for 48 h; (B-1) U251 cells; (B-2) U251 cells treated with TMZ for 48 h; (C-1) U251 cells transfected with livin-shRNA; (C-2) U251 cells transfected with livin-shRNA and treated with TMZ for 48 h; (D-1) livin-overexpressing U251 stem cells; (D-2) livin-overexpressing U251 stem cells treated with TMZ for 48 h; (E-1) U251 stem cells; (E-2) U251 stem cells treated with TMZ for 48 h; (F-1) U251 stem cells transfected with livin-shRNA; (F-2) U251 stem cell transfected with livin-shRNA and treated with TMZ for 48 h.

For U251 stem cells, compared with the respective blank control group, the livin-overexpressing and shRNA groups demonstrated slight increases in the proportion of cells in the S phase of the cell cycle (P<0.05). Following intervention with 400 μmol/l TMZ for 48 h, the blank control group underwent an increase in the S phase, the livin-overexpressing group had marked increases in the proportion of cells in the G0 and G2-M phases and the livin-shRNA group had increases in the S and G2-M phases (P<0.05; Fig. 3).

## Discussion

According to the theory of antiapoptotic gene expression, the proliferation and apoptosis of tumors occurs due to gene expression imbalance (9). Gene expression is controlled by various factors *in vitro* and *in vivo*; the expression of antiapoptotic genes causes a reduction of cell apoptosis, eventually leading to malignant cell proliferation. Therefore, the investigation of methods for the effective induction of cell apoptosis in order to cure cancer have become a focus for numerous studies. Livin (also known as KIAP or ML-IAP) is a member of the apoptosis suppressor protein (inhibitor of apoptosis protein; IAP) family. Among the eight members of the IAP family, only livin has two subunits (α and β); thus, livin has a stronger antiapoptotic effect compared with the other members (10). It plays a key role in cell apoptosis and proliferation, and the cell cycle (11). Members of the IAP family have a repetitive BIR structural domain and/or a RING domain. A previous study demonstrated that the antiapoptotic mechanisms of the IAP family mainly involve the direct interaction of the BIR structural domain with a combination of caspases 3 and 8, which blocks caspase activation and prevents apoptosis (12). Since 2005, our group have observed that the overexpression of livin in U251 glioma cells and the associated stem cells blocks the antiapoptotic induction channel, restricting the transduction of death signals, and has a close relationship with chemotherapy resistance (7,13,14). Results have indicated that livin and caspase are closely related, as they play important roles in apoptotic and anti-apoptotic processes, respectively. The expression of livin was inhibited more strongly in the U251 cells than in the U251 stem cells, which indicates that stem cells have a stronger resistance to TMZ than U251 cells have under the same conditions.

Caspases are cysteine proteases that regulate apoptosis. They are able to promote apoptosis through the protease cascade reaction. Caspase proteins may be divided into three categories: apoptosis initiators (caspase-9), apoptosis executioners (caspase-3 and -7) and inflammation mediators (15). Livin executes an antiapoptotic effect mainly through controlling the cascading activation reaction regulated by combined caspase proteins. Nachmias found that livin combines with caspase-9, thereby exerting an antiapoptotic effect in the initial phase of apoptosis (12). Caspase family proteases are the key proteases for promoting apoptosis. They may be activated both by the death receptor and mitochondrial-mediated cell apoptosis pathways. In particular, caspase-3-mediated apoptosis is the key mechanism of cell apoptosis (16). Activated caspase proteins have been shown to hydrolyze a large number of molecular proteins in cells, and finally lead to cell death, as indicated by both biological chemistry and morphological analysis (12). In the present study, the expression of caspase-3 increased as the TMZ concentration and TMZ intervention time increased. Cell apoptosis was consequently induced, which caused the number of tumor cells to decrease and inhibited tumor growth.

TMZ is a commonly used chemotherapy agent for the treatment of glioblastoma multiforme. The mechanism of the cytotoxic effect of TMZ is mainly through the methylation of the guanine O^6^ position of the DNA repair protein O^6^-alkylguanine DNA alkyltransferase (MGMT), which changes its structure and reduces its activity (17). Although TMZ is able to increase the 2-year survival rate of patients significantly, long-term survivors are seldom found. The biological characteristics of cancer stem cells may be the cause of the failure of long-term chemotherapy; MRPs are expressed in the cancer stem cells isolated from certain solid tumors, and may provide the tumor progenitor cells with resistance to the killing effect of cytotoxic drugs and alter the differentiation of cells. TMZ may efficiently inhibit cell proliferation rather than induce cell death in cancer stem cells (18). The study by Beier *et al* provided the important evidence that CD133^+^ cancer stem cells display resistance to conventional chemotherapy drugs; the CD133^+^ levels of recurrent tumors are higher than those of primary tumors in patients with glioblastoma multiforme (19). Overmeyer *et al* reported that TMZ caused the aging and apoptosis of glioblastoma multiforme cells, and that mutations of tumor suppressor genes, such as P53, could reduce the sensitivity of the cells to TMZ-induced apoptosis. There is also certain evidence indicating that TMZ may overcome the resistance of glioblastoma multiforme to apoptosis by inducing autophagy (20). In the present study, following TMZ intervention, the results showed that TMZ inhibited the apoptosis process by inhibiting the expression of livin, increasing the expression of caspase-3 and arresting the cell cycle.

Based on the above observations and the previous study results, by using lentiviral transfection technology, cell models including overexpression, natural expression and silenced expression of livin were successfully constructed. The results demonstrated that livin plays an important role in the process of cell proliferation; the higher the expression level of livin, the faster cells proliferate. Following TMZ intervention, it was found that the mechanism of impact on the cell cycle differed between cancer stem cells and normal cells with the same intervention. The U251 cells stagnated in the G2-M phase, whereas the U251 stem cells stagnated in the S phase. The expression levels of caspase-3 increased as the concentration of TMZ increased. Caspase-3 may accelerate apoptosis and has a certain relationship with the expression of livin; however, the mechanism is not yet clear and requires further study.

## Figures and Tables

**Table I:** mRNA expression levels of livin prior to and following treatment with various concentrations of TMZ for 48 h (mean ± SD).

		TMZ concentration (μmol/l)					
		
Livin status	Cells	0	25	50	100	200	400
OE (10^−3^)	ACC	0.441±0.025^c^	0.425±0.027	0.294±0.021^b^	0.264±0.017^b^	0.202±0.022^b^	0.105±0.016^b^
	CSC	8.364±0.56^c^,^d^	6.506±0.419^a^,^d^	6.439±0.437^b^,^d^	6.193±0.354^b^,^d^	4.744±0.283^b^,^d^	4.386±0.416^b^,^d^
CON (10^−5^)	ACC	2.137±0.345	0.055±0.012^b^	0.027±0.002^b^	0.021±0.006^b^	0.016±0.002^b^	0.013±0.002^b^
	CSC	50.025±3.182^d^	27.230±3.294^b^,^d^	22.403±2.686^b^,^d^	11.581±2.740^b^,^d^	10.865±2.917^b^,^d^	9.428±2.503^b^,^d^
shRNA (10^−7^)	ACC	2.236±0.196^c^	1.208±0.165^b^	1.203±0.168^b^	1.016±0.015^b^	0.084±0.013^b^	0.059±0.019^b^
	CSC	7.399±0.760^c^,^d^	5.747±0.625^a^,^d^	4.480±0.484^b^,^d^	3.550±0.487^b^,^d^	2.455±0.421^b^,^d^	1.700±0.342^b^,^d^

ACC, autologous cancer cells; CSC, cancer stem cells; OE, livin overexpression group; CON, control group; shRNA, livin short hairpin RNA group; TMZ, temozolomide.

a P<0.05,

b P<0.01 vs. the same cells treated with 0 μmol/l;

c P<0.01 vs. CON in same cell type treated with 0 μmol/l;

d P<0.01 vs. ACC with the same drug concentration and livin status.

**Table II:** mRNA expression levels of caspase-3 prior to and following treatment with various concentrations of TMZ for 48 h (mean ± SD).

		TMZ concentration (μmol/l)					
		
Livin status	Cells	0	25	50	100	200	400
OE (10^−5^)	ACC	0.479±0.054^c^	0.647±0.050^a^	0.997±0.199^a^	1.203±0.281^a^	1.646±0.388^b^	1.772±0.394^b^
	CSC	1.111±0.270^c^,^e^	1.794±0.417^f^	4.760±0.513^b^,^f^	6.274±0.501^b^,^f^	6.349±0.464^b^,^f^	9.677±0.689^b^,^f^
CON (10^−5^)	ACC	1.145±0.316	1.454±0.478	2.133±0.547	2.841±0.680^a^	3.918±0.604^b^	5.632±0.607^b^
	CSC	2.083±0.392^e^	3.473±0.466^a^,^f^	4.636±0.587^b^,^f^	4.740±0.503^b^,^e^	5.179±0.518^b^	8.052±0.745^b^,^e^
shRNA (10^−5^)	ACC	3.276±0.504^d^	3.188±0.500	3.559±0.451	4.461±0.501^a^	6.471±0.452^b^	6.807±0.518^b^
	CSC	16.525±1.825^d^,^f^	22.421±2.151^a^,^f^	27.521±2.371^b^,^f^	37.191±3.160^b^,^f^	53.518±4.055^b^,^f^	69.564±5.538^b^,^f^

ACC, autologous cancer cells; CSC, cancer stem cells; OE, livin overexpression group; CON, control group; shRNA: livin short hairpin RNA group; TMZ, temozolomide.

a P<0.05,

b P<0.01 vs. the same cells treated with 0 μmol/l;

c P<0.05,

d P<0.01 vs. CON in the same cell type treated with 0 μmol/l;

e P<0.05 and

f P<0.01 CSC vs. ACC with the same drug concentration and livin status.

**Table III:** mRNA expression levels of livin and caspase-3 following treatment with 400 μmol/l TMZ for various times (data presented as mean ± SD).

		Time			
		
mRNA	Cells	0 h	24 h	48 h	72 h
Livin (x10^−5^)	ACC	2.464±0.111	0.014±0.002^a^	0.013±0.002^a^	0.007±0.001^a^
	CSC	54.438±2.16^b^	9.842±0.261^a^,^d^	9.428±0.253^a^,^d^	1.740±0.202^a^,^d^
Caspase-3 (x10^−5^)	ACC	1.157±0.091	5.031±0.422^a^	5.632±0.607^a^	8.246±0.4811^a^
	CSC	2.132±0.127^b^	5.357±0.466^a^	8.052±0.747^a^,^c^	11.081±1.450^a^,^c^

ACC, autologous cancer cells; CSC, cancer stem cells.

a P<0.01 vs. the control (0 h) group for the same cells;

b P<0.01 vs. ACC in the control (0 h) group;

c P<0.05,

d P<0.01 vs. ACC with the same treatment time.
